# Effectiveness of tutor shadowing on faculty development in problem-based learning

**DOI:** 10.1186/s12909-022-03615-0

**Published:** 2022-07-22

**Authors:** Chiao-Ling Tsai, Yen-Lin Chiu, Chia-Ter Chao, Mong-Wei Lin, Chao-Chi Ho, Huey-Ling Chen, Bor-Ching Sheu, Chiun Hsu, Chih-Wei Yang

**Affiliations:** 1 Division of Radiation Oncology, Department of Oncology, National Taiwan University Hospital, Taipei, Taiwan; 2 Center of Faculty Development and Curriculum Integration, National Taiwan University College of Medicine, Taipei, Taiwan; 3 Graduate Institute of Medical Education and Bioethics, National Taiwan University College of Medicine, No. 1, Sec. 1, Ren’ai Rd., Zhongzheng Dist., 100 Taipei, Taiwan; 4 Nephrology Division, Department of Internal Medicine, National Taiwan University Hospital, Taipei, Taiwan; 5 Department of Surgery, National Taiwan University Hospital, Taipei, Taiwan; 6 Chest Medicine Division, Department of Internal Medicine, National Taiwan University Hospital, Taipei, Taiwan; 7 Department of Pediatrics, National Taiwan University Hospital, Taipei, Taiwan; 8 Department of Obstetrics and Gynecology, National Taiwan University Hospital, Taipei, Taiwan; 9 Department of Education and Research, Department of Medical Oncology, National Taiwan University Cancer Center, Taipei, Taiwan; 10 Department of Medical Education, National Taiwan University Hospital, Taipei, Taiwan; 11 Department of Emergency Medicine, National Taiwan University Hospital, Taipei, Taiwan

**Keywords:** Tutor shadowing, Faculty development, Problem-based learning

## Abstract

**Background:**

To enhance tutors’ teaching skills, tutor shadowing for novice tutors of problem-based learning (PBL) in addition to conventional faculty development (FD) was applied. This study aimed to develop a tutoring-skill scale (TS-scale) and evaluate the effect of shadowing on PBL tutors.

**Methods:**

This study employed a before-and-after study design with three phases. In phase 1, a TS-scale was elaborated. A validity examination was performed in phase 2. Phase 3 was a study of the effectiveness using a TS-scale survey of novice PBL tutors before and after the FD course. The FD course for novice PBL tutors included an FD workshop and PBL shadowing activities.

**Results:**

A TS-scale with a 32-item questionnaire of self-rated confidence for PBL tutors was identified in phase 1. In phase 2, 7 experienced specialists in medical education were invited to evaluate the content validity of the scale. The item content validity index (I-CVI) ranged from 0.86 to 1, and the scale-CVI (S-CVI) was 0.95. A total of 85 novice PBL tutors completed the TS-scale before the FD course, yielding a Cronbach’s alpha of 0.98. An exploratory factor analysis with varimax rotation was performed. The twenty-four items with significant loadings greater than 0.5 were incorporated into a new TS-scale and were grouped into three factors: student contact, medical expertise, and teaching expertise. In phase 3, 76 novice PBL tutors completed the 24-item TS-scale before (pretest) and after (posttest) the FD course. Their self-rated confidence improved significantly across the three factors after the FD course. The pretest and posttest scores did not differ according to the tutors’ gender, the grades they taught, or their specialty background.

**Conclusions:**

Novice PBL tutors benefit from FD that incorporates tutor shadowing in the 3 key domains of tutoring competencies. The TS-scale developed in this study can be applied in future research on FD design.

**Supplementary Information:**

The online version contains supplementary material available at 10.1186/s12909-022-03615-0.

## Background

The recruitment and training of tutors are necessary when developing a problem-based learning (PBL) curriculum. In PBL, students usually solve an open-ended problem with peers. The tutors drive toward a possible solution with students instead of imparting knowledge [1]. For medical students, PBL curricula are composed of clinical scenarios targeting learning goals. Medical students learn through a collaborative problem-solving process [2]. PBL tutors are expected to promote student-centred learning and drive the discussion toward the lesson’s objectives. Using basic scientific techniques, PBL tutors help guide students and bridge the gaps in clinical subjects. Ultimately, PBL tutors must assess learning outcomes and provide feedback. To lead a successful PBL session, tutors must have strong facilitation skills, which are difficult to improve through conventional faculty development [3].

The skills needed for tutoring in PBL curricula differ from those needed for teaching in a lecture-based program. There is a consensus on the importance of faculty development (FD) for PBL tutors to assist in their responsibilities and roles [4]. The tutors can become proficient facilitators and activators through PBL training programs [1]. However, a lecture-based FD is unable to fill the gap between facilitating theory and actual practice. Most of the effective PBL FD for tutors includes novel designs, such as direct observation, video clips, interactive film, scene scenarios, and role-plays [5, 6]. Situated learning, including tutor shadowing during teaching, is mandatory for PBL tutors.

Tutor shadowing, also known as peer observation, is an activity that involves inspecting colleagues’ teaching practices. The teachers can learn from each other and achieve professional growth. Tutor shadowing is gradually becoming a feature of higher education practice and can be categorized into three basic models: evaluation, development, and peer review [7]. The goal of shadowing is to exchange and reflect on personal methods of teaching. Colleagues can work together to perfect their teaching approach and identify areas where they need to improve. For academic units, peer observation improves the quality of both education and instruction [8]. Tutors have to actively participate in the students’ self-directed learning processes while following the PBL curriculum. To improve facilitating abilities in PBL tutoring, the literature indicates that the direct observation of PBL courses led by experienced tutors may be helpful [9]. To reinforce the FD of PBL, the National Taiwan University College of Medicine (NTUCM) adopted tutor shadowing for novice tutors in addition to lecture-based workshops.

In this study, the elaboration of a modified tutoring-skill scale (TS-scale) for measuring the facilitation skills of PBL tutors was addressed. The TS-scale was validated to assess the effects between conventional FD activity and tutor shadowing on the skills of novice tutors.

## Methods

### PBL of NTUCM

In NTUCM, PBL was first incorporated into the medical curriculum in 1993. The topics of the PBL class emphasize “humanity and society” for second-year medical (M2) students, “anatomy and physiology” for M3 students, and “pathology and pharmacology” for M4 students. In each PBL class, there are 8 to 11 students and a tutor to conduct the class, which lasts 2 h per week. The discussion topics are composed of several clinical scenarios with a preset schedule provided by the course administrator at the beginning of each semester. For example, the discussion scenarios for M2 students include professional norms, medical ethics, medical insurance, laws and regulations, stigmatization, vulnerable people, etc. The PBL cases comprise clear objectives, and paragraphs outlining the real clinical scenarios with problems and references. Before each session, the student self-studies the relevant literature according to the assignment and formulates his/her own knowledge required for problem-solving. The students might share the preparatory work before PBL class, present it on-site with slides, or write it down on the whiteboard directly. Some groups have course leaders during their PBL sessions, and some do not. Every student needs to express his/her own viewpoints on the learning objectives. The students would address their observations, raise questions, brainstorm, and propose some solutions for the issues to develop teamwork and problem-solving skills [10]. During class, the facilitator guides the seven steps in PBL [11]. The tutors promote the proper path for PBL discussion by aiding students to define and analyze the problem by asking open-ended questions. The students are steered to formulate learning objectives, collect additional information and synthesize and test the newly acquired information [12]. Over the years, students’ learning strategies for PBL have changed from offline searches to online surveys. Among the students, the preparative discussion is held on social media rather than as face-to-face conversations as in previous years. The majority of PBL classes remain on campus courses that involve tutors.

### Study design

#### Recruitment of tutors

In the NTUCM, the qualified PBL tutors are attending physicians from the health care system of the National Taiwan University Hospital (NTUH) who volunteer to participate in tutoring. PBL tutors from different specialty backgrounds join a coordinated FD program and learn how to become facilitators. The classic FD course at NTUCM consists of workshops or seminars aimed at teaching and learning strategies for novice PBL tutors. From 2018, NTUCM incorporated the tutor shadowing of PBL classes into the FD of novice tutors.

### FD course description

The FD course for PBL tutors consisted of an FD workshop and tutor-shadowing activities. The FD workshop was composed of a one-day agenda with two panels. In the first panel, there were several lecture-based sessions about educational theory, advances in medical education, and professional development for educators. The second panel were group discussions divided according to which grade the tutors taught. The subjects included pedagogical and content training, PBL facilitation skills, and evaluation. During tutor-shadowing activities, each of the novice tutors joined 2 PBL discussions, which were randomly selected from among the medical PBL courses. The novice tutors joined the PBL classes only as observers. Before the shadowing activity, the observed tutor would introduce the steps of their group discussion to the observer. The observing tutor engaged in the observation exercise silently by investigating the student-centred learning process and the role of facilitating tutors. At the end of the shadowing activity, the observer and the observed had the chance to reflect and give feedback. They might exchange the practice of how their tutorial groups functioned. Afterward, tutors were encouraged to share their impressions from the shadowing activity by email or on tutor forums.

### Study procedure

This study employed a before-and-after study design after a scale development process to explore the effectiveness of FD courses incorporating tutor-shadowing activities [13]. This research included three phases: Phase 1 was TS-scale item elaboration, phase 2 was an examination of validity, and phase 3 was a study of effectiveness.

### Phase 1: literature review and item elaboration

The TS-scale elaboration process followed the recommendations of Hinkin [14]. A literature review on PBL and collected pre-existing scales were performed. Items were generated and modified according to the data from extensive investigations.

### Phase 2: Examination of validity

#### Content validity by professional commentary

The commentary questionnaire of the TS-scale was submitted to experts in the field of medical education to provide content validity. Experts checked for statements and correspondence between the expression and conception of the items. Items from the shadowing scale for experts were scored on a Likert 4-point scale ranging from 1 to 4, with 4 as very appropriate, 3 as appropriate, 2 as inappropriate, and 1 as very inappropriate. The recommendations of the experts were accordingly adopted when revising the questionnaire. The content validity indices (CVI) are reported as item-level (I-CVI) or scale-level (S-CVI) [15, 16]. An I-CVI of 0.78 or higher for three or more experts is considered to indicate agreeable content validity. An S-CVI of 0.80 or higher is considered reasonable.

#### Internal consistency

A group of novice PBL tutors was invited to complete the 32-item TS-scale based on self-rated confidence before the FD activity. The reliability of the TS-scale on internal consistency was tested by Cronbach’s alpha. A Cronbach’s alpha value above 0.8 was considered acceptable [17].

#### Construct validity EFA

Concerning validity analysis based on the theoretical construct, an exploratory factor analysis (EFA) was performed. A new formation of the TS-scale was validated and tailored according to the EFA results.

### Phase 3: study of effectiveness

Qualified novice PBL tutors who participated in the training and completed the validated TS-scale (24 items) with self-rated confidence before (pretest) and after (posttest) the FD course were included for analysis.

### Statistical analysis

A descriptive analysis for participant characteristics was conducted. The Cronbach’s alpha coefficient was calculated for the internal consistency of the scales in our sample. We obtained the Kaiser–Meyer–Olkin (KMO) index and conducted the Bartlett sphericity test to explore the sampling and data adequacy. The EFA using the maximum likelihood method and varimax rotation following the recommended standards was performed [18]. Items with loading values greater than 0.5 were retained. Changes in self-rated confidence before and after tutor shadowing with a paired t test were compared. A *p* value of less than 0.05 was considered statistically significant. All statistical analyses were executed using SPSS (Statistical Package for the Social Sciences) 20.0.

## Results

### Phase 1: TS-scale items elaboration

A literature search of the PubMed database (from August 1, 1990, through July 31, 2016) with the following medical subject heading terms and/or words in the main text: “problem-based learning,” “novice,” “facilitators,” and “questionnaire.” A comprehensive assessment scale for PBL tutors by Slattery and Douglas was identified [19]. The scale contains 32 items with a 5-point Likert scale to assess the self-rated confidence of tutors. The highest score (5) indicated the highest level of agreement on the item measured, and the lowest score (1) represented the lowest level of agreement on the item rated. The items can be categorized by four competencies, namely, (i) facilitation skills, (ii) programme/curriculum knowledge, (iii) personal qualities, and (iv) subject-matter expertise (Table 1).

**Table 1:** Questionnaire items and mean ratings of the self-rated confidence of tutors before tutor-shadowing (TS)

Questionnaire Item^a^	Mean	SD
*Facilitation skills*		
Q1. Ensure group runs well	3.94	0.62
Q2. Monitor participation	4.01	0.66
Q3. Support group identification of learning needs	3.92	0.68
Q7. Feedback group process	4.09	0.61
Q8. Monitor group cohesion	3.75	0.75
Q16. Observe group not participate	4.07	0.65
Q18. Stimulate prior knowledge	3.73	0.82
Q21. Cultivate respect for group opinions	3.93	0.72
Q22. Monitor group agreement	3.91	0.61
Q23. Evaluate group progress	3.91	0.68
Q24. Model knowledge can change	4.01	0.68
Q25. Support use range of resources	3.92	0.71
Q27. Seek range of alternative solutions	3.93	0.69
Q29. Activate students prior experiences	3.74	0.73
Q30. Seek clarification of ideas	3.87	0.69
Q31. Clarify inconsistencies in problem solving	3.85	0.73
Q32. Encourage consideration of range of issues	3.96	0.76
*Programme/curriculum knowledge*		
Q5. Assess students	3.69	0.71
Q6. Liaise between curriculum team and students	3.76	0.78
Q20. Check learning outcomes are achieved	3.92	0.73
*Personal qualities*		
Q11. Role model	3.98	0.67
Q12. Mentor	3.88	0.73
Q13. Work as colleague in group	3.81	0.73
Q14. Share professional experiences	4.31	0.58
Q17. Evaluate own performance	3.74	0.74
*Subject-matter expertise*		
Q4. Explain misunderstandings in knowledge	4.04	0.61
Q9. Give extra information	3.87	0.65
Q10. Offer content knowledge	4.01	0.59
Q15. Identify learning needs	3.82	0.68
Q19. Identify when problem solving is correct	3.82	0.68
Q26. Provide theory if not identified	3.88	0.73
Q28. Direct problem solving	3.99	0.66

^a^Items were adopted from the questionnaire proposed by Slattery and Douglas (2014) [17]

### Phase 2: validity examination of the TS-scale

#### Content validity of the TS-scale

The content validity of the questionnaire was assessed by seven experienced specialists in medical education. According to the experts, all 32 original items were relevant to the study design. The mean score of appropriation from the original 32 items ranged from 3.2 to 4 (mean 3.7). The I-CVI of the original 32 items ranged from 0.86 to 1, and the S-CVI was 0.95, indicating reasonable content validity.

### Internal consistency of the TS-scale

A total of 85 qualified PBL tutors completed the TS-scale before the FD course. To evaluate the reliability of the scale, Cronbach’s alpha coefficients were calculated. The alpha coefficient for the total scale was.98. The alpha values for the four key tutoring facilitation competencies, (i) facilitation skills, (ii) programme/curriculum knowledge, (iii) personal qualities, and (iv) subject-matter expertise, were 0.96, 0.84, 0.86, and 0.91, respectively. The Cronbach’s alpha coefficients achieved reliable internal consistency estimates for the scale.

### Construct validity of the TS-scale

The appropriateness of the dataset for EFA was examined by estimating the KMO and conducting Bartlett’s test of sphericity. The KMO measure of the sampling adequacy was 0.91. Bartlett’s test was statistically significant at *p* < 0.001. The results suggested adequacy sampling for conducting the factor analysis [20]. The cut-off value of 0.5 was commonly used to determine an acceptable factor loading [21, 22]. Thus, 24 items with loadings greater than 0.5 were selected for the TS-scale. According to the Kaiser rule, the factors with eigenvalues greater than 1 were considered significant [20]. Finally, the 24 items could be grouped into three factors: student contact, medical expertise, and teaching expertise. The total variance explained by the three factors reached 69.06% (Table 2).

**Table 2:** The results of exploratory factor analysis

	Item	Factor1	Factor2	Factor3
**Student contact**				
Q18	Stimulate prior knowledge	0.78		
Q20	Check learning outcomes are achieved	0.77		
Q29	Activate students prior experiences	0.77		
Q19	Identify when problem solving is correct	0.75		
Q6	Liaise between curriculum team and students	0.71		
Q5	Assess students	0.69		
Q23	Evaluate group progress	0.66		
Q8	Monitor group cohesion	0.66		
Q17	Evaluate own performance	0.63		
Q22	Monitor group agreement	0.60		
Q21	Cultivate respect for group opinions	0.56		
**Teaching expertise**				
Q11	Role model		0.79	
Q12	Mentor		0.76	
Q13	Work as colleague in group		0.72	
Q16	Observe group not participate		0.64	
Q2	Monitor participation		0.63	
**Medical expertise**				
Q10	Offer content knowledge			0.86
Q4	Explain misunderstandings in knowledge			0.81
Q14	Share professional experiences			0.63
Q32	Encourage consideration of range of issues			0.58
Q30	Seek clarification of ideas			0.57
Q9	Give extra information			0.57
Q31	Clarify inconsistencies in problem solving			0.54
Q24	Model knowledge can change			0.49
KMO = 0.913				
Bartlett’s Test of Sphericity = 1799.914				
Eigenvalues		7.04	4.93	4.60
Variance explanation (%) = 69.056		58.10	6.33	4.62

### Phase 3: study of effectiveness

#### Characteristics of participants

A total of 76 novice PBL tutors from the academic years 2018 to 2020 from 17 specialty backgrounds were recruited. The majority of tutors were men (77.6%). Twenty-two (28.9%) tutors, 24 (31.6%) tutors, and 30 (39.5%) tutors observed the PBL class for the M2 students, M3 students, and M4 students, respectively. The backgrounds and characteristics of the tutors are summarized in Table 3. Participants completed the questionnaire based on their self-rated confidence before the tutor shadowing activities (Table 1).

**Table 3:** Background and characteristics of tutors

Features	*N* = 76
	**No (%)**
**Gender**	
Men	59 (77.6%)
Women	17 (22.4%)
**Category by school years**	
2nd year (humanity/society)	22 (28.9%)
3rd year (anatomy/physiology)	24 (31.6%)
4th year (pathology/pharmacology)	30 (39.5%)
**Specialty**	
Internal Medicine	22 (28.9%)
Surgery	10 (13.2%)
Oncology	9 (11.8%)
Orthopedic Surgery	7 (9.2%)
Neurology	5 (6.6%)
Radiology	4 (5.3%)
Anesthesiology	3 (3.9%)
Nuclear Medicine	3 (3.9%)
Geriatrics	2 (2.6%)
Obsterics and Gynecology	2 (2.6%)
Ophthalmology	2 (2.6%)
Pediatrics	2 (2.6%)
Physical Medicine and Rehabilitation	2 (2.6%)
Family Medicine	1 (1.3%)
Otolaryngology	1 (1.3%)
Pathology	1 (1.3%)
Urology	1 (1.3%)

### The effectiveness of the tutor shadowing activities

**Fig. 1:**
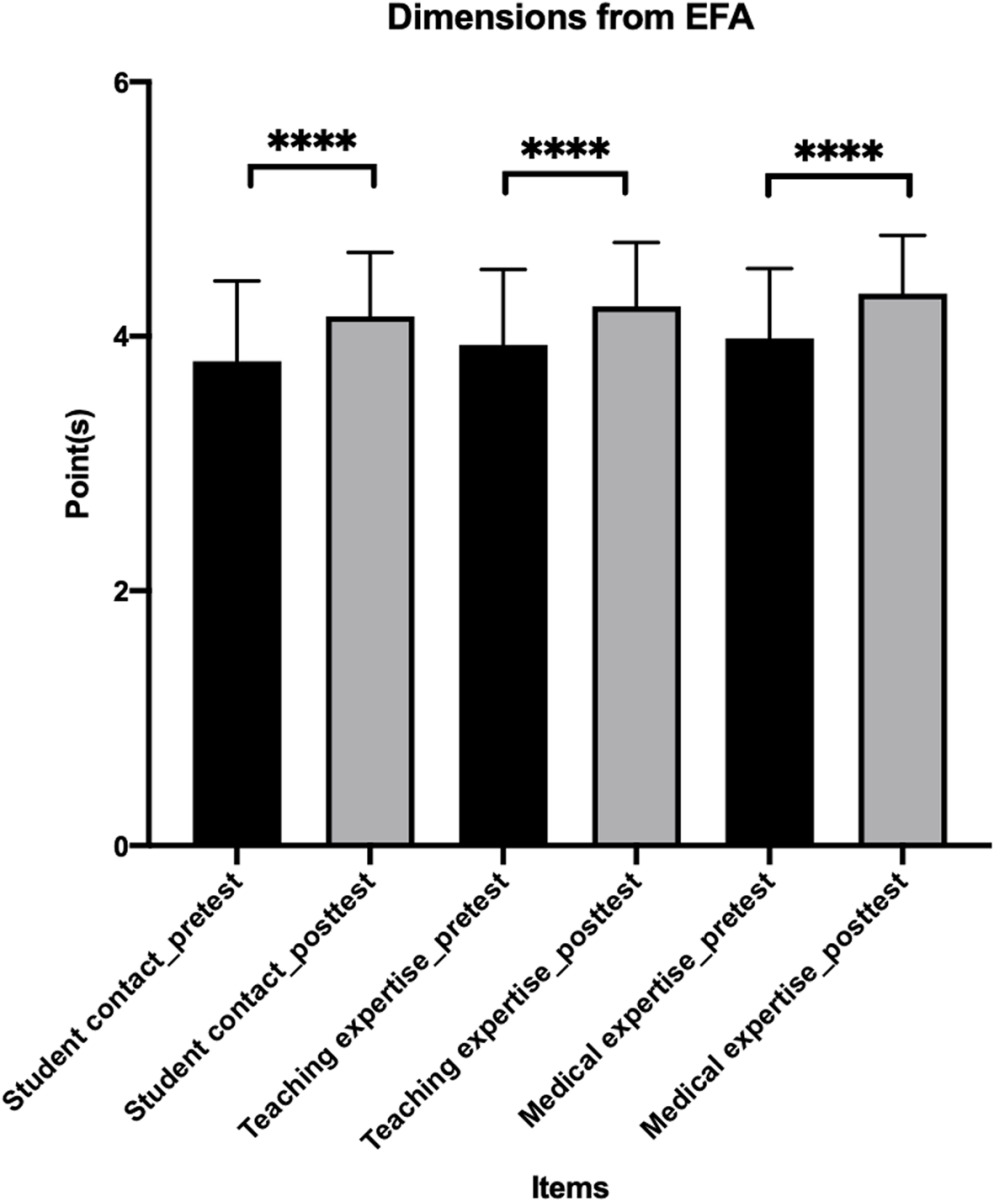
The effectiveness of tutor shadowing for novice PBL tutors. ****: *p* < 0.001; PBL: problem-based learning; EFA: exploratory factor analysis

The 76 novice PBL tutors completed the training course. The responses before (pretest) and after (posttest) the training course were used to evaluate the effectiveness of the FD course. A newly formed TS-scale was created according to the EFA results and was used after validation. The Cronbach’s α coefficients for the three factors of student contact, teaching expertise and medical expertise were 0.95, 0.89, and 0.91, respectively. The self-rated confidence improved significantly after the FD across the same three factors, with pretest and posttest values of 3.8 ± 0.6 and 4.2 ± 0.5 with *p* < 0.001 for student contact, 3.9 ± 0.6 and 4.2 ± 0.5 with *p* < 0.001 for medical expertise, and 4.0 ± 0.5 and 4.3 ± 0.5 with *p* < 0.001 for teaching expertise (Fig. 1). The pretest and posttest scores did not differ according to the tutors’ gender, the grade they taught, or their specialty background (Fig. S1).

## Discussion

Applying a robust process, a new TS-scale was successfully developed and validated in our study. The validated TS-scale was composed of 24 items that were grouped into three categories. The scale items were reliable and had adequate internal consistency. In this study, highly representative participants with a variety of specialties who were equally distributed according to the grades they taught were enrolled. These novice PBL tutors were experienced teachers in their medical fields but may be unfamiliar with how to efficiently facilitate a small-group PBL class. A training program incorporating workshops and course observation for novice tutors was implemented with tutor shadowing. With the aid of the validated TS-scale, the advantage of tutor shadowing for novice PBL tutors in terms of 3 key facilitation competency categories was clarified.

PBL has been widely used as a pedagogical strategy in medical education worldwide for decades. There is little guidance available on how to become a successful PBL tutor. Teaching and mentoring are essential components of PBL tutoring and need to be developed through a systemic process [23]. Over the years, the FD of PBL has been reformed, resulting in substantial modifications. FD activities include reflection, educational projects to enhance teaching effectiveness, and formal, structured workshops designed as experiential learning [24]. Nevertheless, tutoring skills in facilitating the process of PBL could be further optimally cultivated via observational learning [25]. Tutor shadowing is one activity that interests medical professionals, fosters peer learning and encourages teamwork. It is a highly personalized, learner-centred method to impel the socialization and professional development of faculty members [26]. Through observing the PBL curriculum with an experienced tutor, the observer tutor has the chance to acquire the facilitation skill for PBL directly.

A recent review found that most FD programs provide excellent satisfaction and result in positive changes in teaching [24]. These evaluations of the FD programs depended on ascertained, self-assessments instead of observing and judging teaching practice [23]. However, the majority of the assessments were not validated. A tailored questionnaire is essential for the quality improvement of individual FD programs. To evaluate the behaviour change in teaching skills, a performance-based measure of change is necessary [27]. Multiple methods and outcome measures would support the development of future FD [24]. The current evaluation instrument for tutor shadowing focuses on peer feedback and strategies for instructional practice [28]. A convenient instrument that could be easily implemented to value the effectiveness of shadowing activity is noteworthy. Quantification is a prerequisite for agreeing upon setting benchmarks [29]. In an attempt to determine the impact of shadowing on PBL with scale, the FD questionnaire for novice PBL tutors proposed by Slattery et al. was adopted in our study [19]. The 3-phase study design was utilized to reformulate and validate the scale with 24 items. From our study, the new TS-scale was proved to be usable and applicable to FD of tutoring shadowing for PBL tutors. The new TS-scale is easy to use and efficient.

The expert observation of teaching is a key element of FD in higher education and provides teaching practice and boosts confidence [30]. Similarly, the majority of medical doctors learned to be educators by observing the teaching practices of a senior faculty member in a clinical scenario. The incorporation of peer observation into tutor training in PBL curriculum is practicable. The peer observation of teaching with group feedback supports the development of teaching competencies [31]. Garcia et al. used video-recorded PBL sessions for self-observation and peer feedback as an FD approach for PBL tutors [28]. Self-observation strengthens awareness and cultivates student learning. Peer coaching helps tutors facilitate processes in PBL sessions. Informal teacher communities also enhance the professional development of PBL tutors [32]. To support tutoring practice, NTUCM introduced tutor shadowing in 2018. Most tutors agreed in the qualitative interviews that the tutor shadowing made a positive contribution to their development as tutors. From the commentary collected directly from tutors, emails, and tutor forums, tutors acknowledged the merit to learn from peers. Through shadowing, they had the chance to gain new insights into employing cognitive strategies, how to facilitate group dynamics, and provide structured feedback during PBL discussion. To objectively evaluate the effect of shadowing on tutoring, a quantitative scale was developed in this study.

In the 1990s, FD for PBL included general skill, developmental, comprehensive, and course-based models [25]. With the integration of adult learning theory into FD for PBL tutors, new models of tutor training continued to be created [33]. Program evaluation and outcome-based studies have also been developed to ensure that PBL curriculum is successful. Harden and Crosby identified twelve roles for the medical teacher [34]. The 12 roles are categorized into teaching expertise, medical expertise, student contact, and students at a distance. According to Harden and Crosby, we arranged our new 24-item TS-scale into student contact, medical expertise, and teaching expertise (Table 3). These aspects are essential components in the PBL class. The tutors’ perception of these three aspects helps us evaluate the effect of FD activities on facilitation skills.

Limitation.

This study has some limitations. First, our tutors were experts in their medical fields and had experience in teaching techniques. The novice tutors had diverse specialty backgrounds. However, we failed to define the individualized benefit of tutor shadowing according to gender or background. The TS-scale has to be examined with more tutors to confirm the conclusions drawn in this study. Second, the results of the TS-scale in our study were based solely on tutors’ self-perceptions. Self-recognized changes in PBL tutoring might not be reflected real behavioural changes. Further studies are needed to confirm the effect of shadowing on tutoring behaviour.

## Conclusions

Tutoring skills are essential in PBL activities. Tutors need to learn to listen, ask inquiry-based questions, and monitor the PBL process. This curriculum mode abandons the traditional role of a teacher as a content expert and knowledge dispenser. Thus, facilitating the development of new tutoring skills while also transforming faculty beliefs about teaching and learning are the dual challenges of PBL FD [25]. Tutor shadowing improves the success of FD for PBL tutors. Based on the results of our study, we found that tutor shadowing comprehensively enhanced three aspects of novice PBL tutors’ work: student contact, medical expertise, and teaching expertise. We were able to use the TS-scale that divided 24 items into 3 dimensions to measure the level of self-rated confidence of novice PBL tutors after tutor shadowing sessions. We believe that the TS-scale from this study can contribute to the evaluation of future FD activity.

## Supplementary Information

**Additional file 1.**

## Data Availability

The datasets used and analyzed during the current study are available from the corresponding author on reasonable request.

## Abbreviations

CVI: content validity indexes
EFA: Exploratory Factor Analysis
FD: faculty development
I-CVI: item-level content validity indexes
KMO: Kaiser-Meyer-Olkin
TS: tutoring-skill
PBL: problem-based learning
NTUCM: National Taiwan University College of Medicine
NTUH: National Taiwan University Hospital
M2: second-year medical students
M3: third-year medical students
M4: fourth-year medical students
S-CVI: scale-level content validity indexes
