# Supplementary material for: Effectiveness of tutor shadowing on faculty development in problem-based learning
Source: BMC Med Educ. 2022 Jul 22;22:564. doi: 10.1186/s12909-022-03615-0 (PMC9306026; doi:10.1186/s12909-022-03615-0)

**Supplemental Information**

**Figure S1. The effectiveness of tutor shadowing for novice PBL tutors by the (A) tutor’s gender, (B) taught grade, and (C) specialty background. PBL: problem-based learning; EFA: Exploratory Factor Analysis; M2: second-year medical students; M3: third-year medical students; M4: fourth-year medical students; ns: non-significant.**


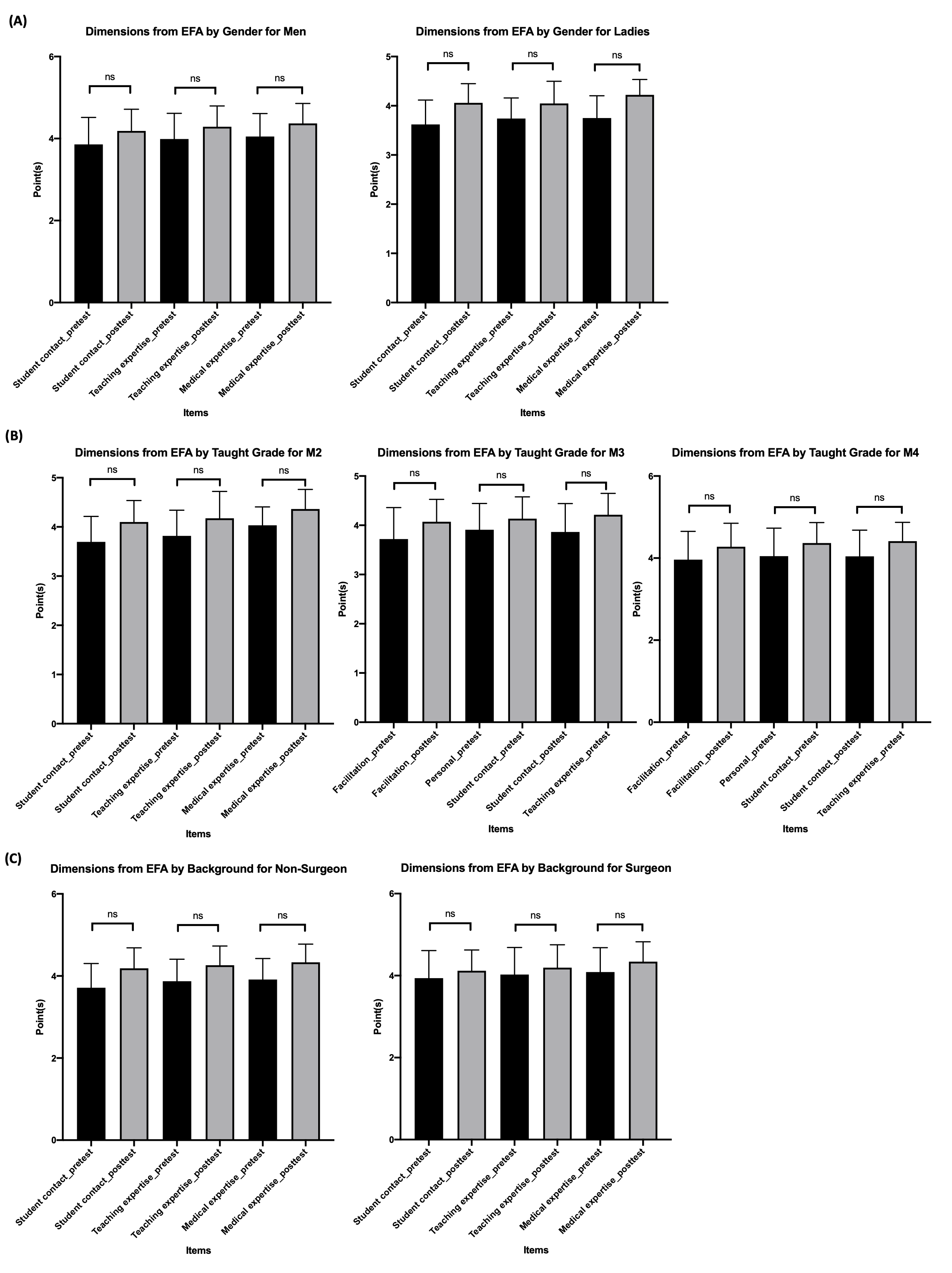

Supplement: Supplementary file 1 — Additional file 1. [file 12909_2022_3615_MOESM1_ESM.docx]
